# Pharmacokinetics of ceftriaxone-tazobactam (8:1) combination in healthy and *Escherichia coli* induced diarrhoeic birds

**DOI:** 10.5599/admet.1170

**Published:** 2022-09-13

**Authors:** U.C. Mithin, Rinku Buragohain, Pradip K Das, Tapan K Mandal, Rabindra N Hansda, Siddhartha N Joardar, Indranil Samanta, Tapas K Sar

**Affiliations:** 1 Department of Veterinary Pharmacology and Toxicology, West Bengal University of Animal and Fishery Sciences, 37 K. B. Sarani, Kolkata-700037, West Bengal, India; 2 Department of Veterinary Physiology, West Bengal University of Animal and Fishery Sciences, 37 K. B. Sarani, Kolkata-700037, West Bengal, India; 3 Department of Veterinary Pathology, West Bengal University of Animal and Fishery Sciences, 37 K. B. Sarani, Kolkata-700037, West Bengal, India; 4 Department of Veterinary Microbiology, West Bengal University of Animal and Fishery Sciences, 37 K. B. Sarani, Kolkata-700037, West Bengal, India

**Keywords:** Disposition, efficacy study, antibiotic-β lactamase inhibitor combination, poultry, intramuscular injection

## Abstract

Antibiotic-resistant *Escherichia coli* infection of poultry causes significant economic losses. Extended spectrum β lactamases (ESBL) producing *E. coli* was inoculated in a broiler, Rhode Island Red and Haringhata Black birds orally at 56×10^8^ c.f.u. mL^-1^ for induction of diarrhoea. Pharmacokinetics of ceftriaxone-tazobactam combination (8:1) was studied following a single intramuscular injection at 28.125 mg kg^-1^ and the combination was administered twice daily to treat such infection. Plasma concentration of both ceftriaxone persisted up to 8 h in experimental birds and maintained an approximate ratio of 8:1 with tazobactam for a period of 2 h, 0.25 h and 0.75 h, respectively in a broiler, Rhode Island Red and Haringhata Black birds. The *K*_el_ was significantly lower in all experimental birds compared to healthy birds. Efficacy study was conducted in diarrhoeic birds by administration of ceftriaxone-tazobactam combination at 28.125 mg kg^-1^ body weight twice daily intramuscularly for three days which caused an increase in specific antibody titre in the broiler on 5^th^ day and in Rhode Island Red birds 10^th^ day. However, Haringhata black birds were inherently showed more resistance towards the infection. The combination of ceftriaxone and tazobactam in the ratio of 8:1 can be an effective treatment to combat ESBL producing *E. coli* infections.

## Introduction

The poultry sector continues to grow rapidly in many parts of the world. The poultry sector is broadly divided into two sub-sectors, one is the highly organized commercial sector with about 80 % of the total market share and the other being the unorganized sector with about 20 % of the total market share in India [1]. The unorganized sector is also referred to as backyard poultry which plays a key role in supplementary income generation and family nutrition to economically weaker sections (EWS) in India. The Rhode Island Red, an American breed of chicken (*Gallus gallus domesticus*) is a backyard dual-purpose poultry reared by the EWS section of people for its egg-laying ability and hardiness. Whereas Haringhata Black is an indigenous poultry breed found in the northern part of North 24 Parganas and southern part of Nadia districts of West Bengal, India. *Escherichia coli* infections have various disease expressions in domestic birds, including salpingitis, synovitis, omphalitis, and/or chronic respiratory disease. Colibacillosis is one of the principal causes of morbidity and mortality in the poultry industry and it is responsible for significant worldwide economic losses [2]. The experimental use of vaccines against *E. coli* has had limited success due to the antigen used and the methods of inactivation and administration. Vaccination of broiler breeding hens with homologous *E. coli* had demonstrated that maternally derived antibodies protected against colibacillosis for only two weeks post-hatching [3,4]. Recommended treatment protocols for *E. coli* infections include antibiotics with a broad spectrum of activity and sulpha drugs. However, isolates of *E. coli* from poultry are sometimes found to be resistant to commonly used antibacterial drugs. Dhillon and Jack reported failure of oxytetracycline treated feed to reduce losses during outbreaks of colibacillosis in commercial caged layers [5]. In another study performed in Spain, Blanco et al. isolated a septicaemic strain of *E. coli* resistant to fluoroquinolones [6]. Extended spectrum β lactamases (ESBLs) are plasmid-mediated β lactamases that have the ability to hydrolyze β lactam antibiotics containing an oxyimino group (e.g. ceftazidime, ceftriaxone, cefotaxime or aztreonam). These ESBLs were most commonly found in *Klebsiella pneumoniae*, but are being increasingly found also in *E. coli*, *Proteus mirabilis* and other members of the Enterobacteriaceae. The vast majority of ESBLs are derivatives of TEM-1 (the common plasmid-mediated β lactamase of organisms such as *E. coli*) or SHV-1 (the common chromosomally mediated β lactamase of *K. pneumoniae*). TEM-1 and SHV-1 can inactivate ampicillin but not the third-generation cephalosporins [7,8]. Such ESBL containing *E. coli* has already been isolated from backyard poultry in India [9,10]. The infections caused by ESBL strains of *E. coli* were often associated with nosocomial epidemics, and no clear association with food consumption or contact with animals was noticed. But, sometimes poultry meat is also consumed without proper boiling, which may be a source of resistant *E. coli* infections in consumers. Hussain et al. reported that prevalence rates of ESBL producing *E. coli* was 46 % among broiler chicken meat and was 15 % among free-range/backyard chicken meat. *E. coli* isolated from broiler and free-range chicken meat exhibited 68 % and 8 % prevalence rates, respectively, for multi-drug resistant *E. coli* [11]. Ceftriaxone is a broad-spectrum cephalosporin with potent activity against Gram-positive and Gram-negative bacteria, including *Enterobacteriaceae*, *Heamophilus influenza*, *Streptococcus pneumonia* and other non-*enterococcal streptococci* [12]. Li et al. studied the pharmacokinetics of ceftriaxone in broiler poultry following single intravenous administration at 50 mg kg^-1^ and determined tissue residue level of ceftriaxone in plasma, liver, kidney, heart, lungs and muscle tissue [13]. Pharmacokinetics study of ceftriaxone in layer poultry following single-dose intravenous and intramuscular administration at 50 mg kg^-1^ showed favourable pharmacokinetic characteristics for its use as an effective antibiotic against septicemic diseases of poultry [14]. Queenan *et al*. reported a minimum inhibitory concentration (MIC) of ≤ 0.12 μg mL^-1^ for ceftriaxone against inoculums of 10^5^ and 10^6^ c.f.u. mL^-1^ of TEM-1 containing *E. coli* [15]. Tazobactam is an inhibitor of a variety of plasmid-mediated β lactamases elaborated by some bacteria. The ceftriaxone and sulbactam (another β lactamase inhibitor) combination was reported to be more effective than ceftriaxone alone for the prevention of mutation in ESBL producing organisms *in vitro* with MPC (mutation prevention concentration) of > 256 μg mL^-1^ for ceftriaxone-sulbactam combination and >512 μg mL^-1^ for ceftriaxone alone [16]. Whereas Payne *et al*. reported that tazobactam has greater β lactamases inhibitory activity than sulbactam [17]. Ceftriaxone-tazobactam combination (8:1) therapy for ESBL producing *E. coli* infection could be an attractive option. In our previous study, we reported the pharmacokinetics of ceftriaxone and tazobactam following intramuscular administration at 25 mg kg^-1^ and 3.125 mg kg^-1^, respectively, in the broiler Haringhata Black and Rhode Island Red poultry [18]. Moreover, ceftriaxone-tazobactam combination (8:1) at 28.1 mg kg^-1^ intramuscularly twice daily for three days in poultry did not alter aspartate transaminase and alanine transaminase activities significantly [19]. Therefore, the present research work was undertaken to study the pharmacokinetic profile of ceftriaxone-tazobactam combination (8:1) in healthy and ESBL *E. coli* infected broiler, Rhode Island Red and Haringhata Black birds following single intramuscular dosing and to evaluate the efficacy of ceftriaxone-tazobactam combination (8:1) against ESBL producing *E. coli* infection.

## Experimental

### Drugs and chemicals

Analytical grade ceftriaxone sodium (purity ≥ 95 %) was obtained from Alembic Limited, Mumbai, India and analytical grade tazobactam was obtained from Sigma Aldrich. All other chemicals used in the study were obtained from E. Merck (India) and Sigma Chemicals Co., USA.

### Experimental birds

A total of eighteen clinically healthy adult poultry (six poultry each of broiler, Rhode Island Red and Haringhata Black) were collected from Instructional poultry farm, West Bengal University of Animal and Fishery Sciences, Kolkata, India. The broiler birds were approximately 5 weeks of age and the Rhode Island Red and Haringhata Black birds were approximately 12 weeks of age. All birds were quarantined for 14 days period prior to the start of the experiment. Six birds of each breed were caged in three separate metabolic cages made of stainless steel and provided with commercial grower feed with *ad libitum* potable drinking water. All the experimental procedures were approved by the Institutional Animal Ethics Committee (IAEC), West Bengal University of Animal and Fishery Sciences, India and conducted as per the ethical guidelines of the Committee for the Purpose of Control and Supervision of Experiments on Animals (CPCSEA), India.

### Pharmacokinetic study

The pharmacokinetic profile of ceftriaxone and tazobactam was studied in six healthy broiler birds (Gr BCT-H), six Rhode Island Red birds (Gr RCT-H) and six Haringhata Black birds (Gr HCT-H) following single intramuscular administration of ceftriaxone-tazobactam combination (8:1) at 28.125 mg kg^-1^. The same six healthy broiler, Rhode Island Red and Haringhata Black birds were used for induction of diarrhoea by oral inoculation of ESBL producing *E. coli* culture after allowing a washout period of 15 days. The pharmacokinetic profile of ceftriaxone and tazobactam was also studied in these diarrhoeic broiler birds (Gr BCT-D), Rhode Island Red birds (Gr RCT-D) and Haringhata Black birds (Gr HCT-D) following single intramuscular administration of ceftriaxone-tazobactam combination (8:1) at 28.125 mg kg^-1^. Blood samples were collected from the collateral wing vein at predetermined time intervals for estimation of each individual drug concentration.

### Efficacy study

For efficacy study, six apparently healthy birds each of broiler (Gr BE), Rhode Island Red (Gr RE) and Haringhata Black (Gr HE) breed were used for induction of diarrhoea following oral inoculation of ESBL producing *E. coli*. Following induction of diarrhoea, ceftriaxone-tazobactam combination (8:1) was administered at 28.125 mg kg^-1^ two times daily (at 12 h interval) intramuscularly for three days. The dosage regimen was calculated on the basis of maintenance of plasma ceftriaxone concentration above MIC level in a pharmacokinetic study. Treatment with a prescheduled dosage regimen of the ceftriaxone-tazobactam combination was employed on 7^th^-day post-inoculation in broiler and Rhode Island Red birds and on 8^th^-day post-inoculation of the second challenge in Haringhata Black birds. All the birds were closely observed during and after treatment up to a period of 1 month.

### Induction of infection with pathogenic E. coli possessing ESBL genes

The pathogenic *E. coli* was isolated from broiler birds in a local poultry farm. The bird was 29 days old and was suffering from diarrhoea followed by death. The strain belonged to O62 serogroup, pathogenic to experimental chickens and possessed the genes for TEM (bla TEM). However, the strain was negative for other ESBL genes such as bla CTX-M and bla SHV. The isolate was maintained at the Department of Veterinary Microbiology, West Bengal University of Animal & Fishery Sciences, Kolkata. For induction of diarrhoea in the experimental broiler, Rhode Island Red and Haringhata Black birds, 56 × 10^8^ c.f.u. mL^-1^ of the bacterial culture was inoculated by oral route. However, Haringhata Black birds did not show any clinical sign following the initial challenge of ESBL producing *E. coli* (TEM-1) sub-culture (56 × 10^8^ c.f.u. mL^-1^). Due to failure of the initial challenge, these birds were again inoculated orally with a higher second dose (112 × 10^8^ c.f.u. mL^-1^ subculture) after 21 days of oral inoculation with 56 × 10^8^ c.f.u. mL^-1^ subculture of ESBL producing *E. coli*

### Antibiotic sensitivity test

The ESBL producing *E. coli* isolates were tested for their sensitivity and resistance to ceftriaxone and ceftriaxone-tazobactam combination by disc diffusion method [20]. The result was interpreted as per the CLSI guidelines or the standard information provided by the manufacturer.

### Collection of samples

Blood samples (2 mL) were collected from the wing vein in heparinized test tubes at 0 (pre-dosing), 0.04, 0.08, 0.25, 0.5, 0.45, 1, 2, 4, 6, 8, and 12 h post-dosing. Plasma was then separated by centrifugation at 3000 rpm for 20 min and stored at 4 °C for pharmacokinetics analysis. Blood samples (1 mL) without anticoagulant were collected from the experimental birds at a pre-inoculation time (0 day) and 3, 6, 9, 12, 15, 18 and 21 days post-inoculation of ESBL producing *E. coli.* The collected blood samples were allowed to clot at 25 °C and the serum was collected in sterile vials. The serum samples were further centrifuged at 2500 rpm for 15 min, to remove residual RBC and it was stored at -20 °C until further analysis.

Faecal samples were collected following oral inoculation of ESBL producing *E. coli* and on 3^rd^ day of ceftriaxone-tazocactam treatment in the induced diarrhoeic birds for the efficacy study.

### Estimation of ceftriaxone in plasma

Ceftriaxone concentration in plasma was estimated with the method of Sar et al. [21]. The mobile phase was prepared with the method mentioned in United States pharmacopoeia (USP). The mobile phase consisted of 44 mL of potassium phosphate buffer (pH 7.0), 4 mL sodium citrate buffer (pH 5.0), 400 mL of HPLC grade acetonitrile, 3.2 g of tetraheptyl ammonium bromide and 552 mL of HPLC grade milipore water. To 1 mL of plasma, 1 mL acetonitrile (HPLC grade) was added for deproteinisation and shaken vigorously for 1 min followed by centrifugation at 5000 rpm for 20 min. The supernatant was collected and filtered through 0.2 μm membrane filter. The sample filtrate volume was adjusted to 1 mL with acetonitrile and 20 μL of the sample was injected into high-performance liquid chromatography (HPLC). Measurement was performed at a wavelength of 254 nm.

### Estimation of tazobactam in plasma

A binary mobile phase consisting of 25 mM potassium dihydrogen phosphate buffer, pH 6.2 and acetonitrile (94:6, v/v) was used for the estimation of tazobactam. Extraction of tazobactam was done with the method of Ocampo et al. To 0.5 mL of plasma sample, 0.5 mL of mobile phase and 3 mL of acetonitrile was added and vortexed for 3 min. The whole mixture was centrifuge at 2000 rpm for 10 min and the supernatant was decanted into a test tube containing 3 mL of dichloromethane. The whole mixture was vortexed for 1 min followed by centrifugation at 3000 rpm for 10 min. 20 μL of the aqueous layer was injected into HPLC for the estimation of tazobactam concentration [22]. Measurement was performed at a wavelength of 210 nm [23].

### Instrumental condition

SHIMADZU LC-20 AT liquid chromatograph coupled with photo diode-array (PDA) detector attached with computer SPD-MXA 10 software was used for the analysis of the drugs. 5μ Luna C18(2); 250 × 4.6 mm (RP) column was used and 1.5 mL min^-1^ flow rate was maintained.

### Pharmacokinetic parameters

Pharmacokinetic parameters of ceftriaxone and tazobactam were determined from the computerized curve fitting programme “PHARMKIT” supplied by the Department of Pharmacology, JIPMER, Puducherry, India. The data obtained from this programme in healthy and diseased birds were analysed for deriving some of the pharmacokinetic parameters as per standard formulae of Baggot [24]. The fitted pharmacokinetic parameters were MRT, *β*, *t*_½_ *β*, AUC, *V*d_area_, Cl_B_, *C*_max_ and *T*_max_ while the extrapolated pharmacokinetic parameters were A, B, *K*_a_, *t*_½_ *K*_a_, *V*d_c_, *V*d_ss_, *K*_12_, *K*_21_, *K*_el_, *f*_c_ and T~P.

### Bacterial colony count

Bacterial colony count of faecal samples was conducted with the standard protocol by Quinn et al. [25]. The cloacal swabs were collected from the studied birds using sterile cotton swabs (HiMedia, India) and placed into a sterile transport medium (Hi-Media, India). The cloacal swabs were stored at 4 °C, and the content was processed for bacteriological enumeration on the same day of collection. The swab content was serially diluted to 10 folds with sterile phosphate buffer saline solution (PBS). From 10^–2^ and 10 ^–4^ dilution, 10 μL was placed on eosin methylene blue (EMB) Agar (HiMedia, Mumbai, India) for *E. coli*. The plates were incubated aerobically at 37 °C for 24 hours. The typical colonies were enumerated in a colony counter (Digital colony counter, LA663, HiMedia, India) and the numbers were expressed as colony-forming units (c.f.u.) per gram.

### i-ELISA

To detect anti *E. coli* antibody in serum samples of diarrhoeic broiler, Rhode Island Red and Haringhata Black bird, plate ELISA was performed based on principles of i-ELISA as per Mockett et al. [26] with some modifications. The original protocol detected the titer at 405 nm. However, the present study detected the titer at 492 nm wavelength.

### Preparation of somatic antigen

The laboratory maintained strain of ESBL producing *E. coli* was revived on a trypticase soy broth (TSA) slant. It was then transferred to TSB and incubated at 30 °C for 24 h. The cells were harvested by centrifugation at 7500 rpm for 25 min at 4 °C. The cell pellets were washed with normal saline solution (NSS) and finally re-suspended in 10 mL of the NSS. Sonication was performed after adding 25 mM of PMSF and 24 mM of EDTA. The bacterial cell suspension in NSS was sonicated on the ice at the amplitude of 50 for 0.5 to 1 min, for each cycle giving 1 min interval in between. The process was repeated by 6-8 cycles. Soluble sonicated extract was centrifuged at 7500 rpm for 25 min at 4 °C. The supernatant was collected and the soluble protein (somatic antigen) was concentrated by sucrose [27].

### Statistical analysis

The data were expressed as mean ± standard error (S.E.). The data were analyzed statistically using GLM (General Linear Model) method, comparison through LSD (least significant difference) and t-test of IBM SPSS statistic, version 21, 2012.

## Results and discussion

### Antibiotic sensitivity test

**Figure 1.**
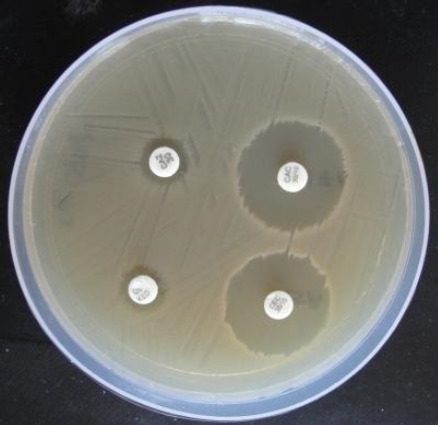
Plate showing sensitivity pattern of only ceftriaxone and ceftriaxone-tazobactam (8:1) combination against ESBL producing *E. coli* during antibiotic sensitivity test.

The ESBL producing *E. coli* isolates showed intermediate sensitivity for ceftriaxone and high sensitivity to ceftriaxone-tazobactam combination (Figure 1). But, the ESBL producing *E. coli* isolates were resistant to ampicillin, amoxicillin, streptomycin, cephalexin and cefotaxime.

### Standardization of the analytical techniques of ceftriaxone and tazobactam

The recovery percentages of ceftriaxone and tazobactam from plasma were 81.14 ± 4.95 % and 83.01 ± 3.6 %, respectively. Since the recovery percentages of both the drugs were more than 80 % in plasma, the recoveries were found to be satisfactory. The limit of detection for both the drugs in plasma was 0.5 ppm and sensitivity was 0.25 ppm. The linearity of calibration curves was checked for both the drugs and linearity was found to be maintained in the range of 0.5 to 25 ppm for ceftriaxone and 0.5 to 10 ppm for tazobactam in plasma. However, the retention time of ceftriaxone and tazobactam showed inter-day variation.

### Pharmacokinetics of ceftriaxone and tazobactam in healthy and ESBL E. coli infected broiler birds following single intramuscular administration of ceftriaxone-tazobactam combination (8:1)

**Figure 2.**
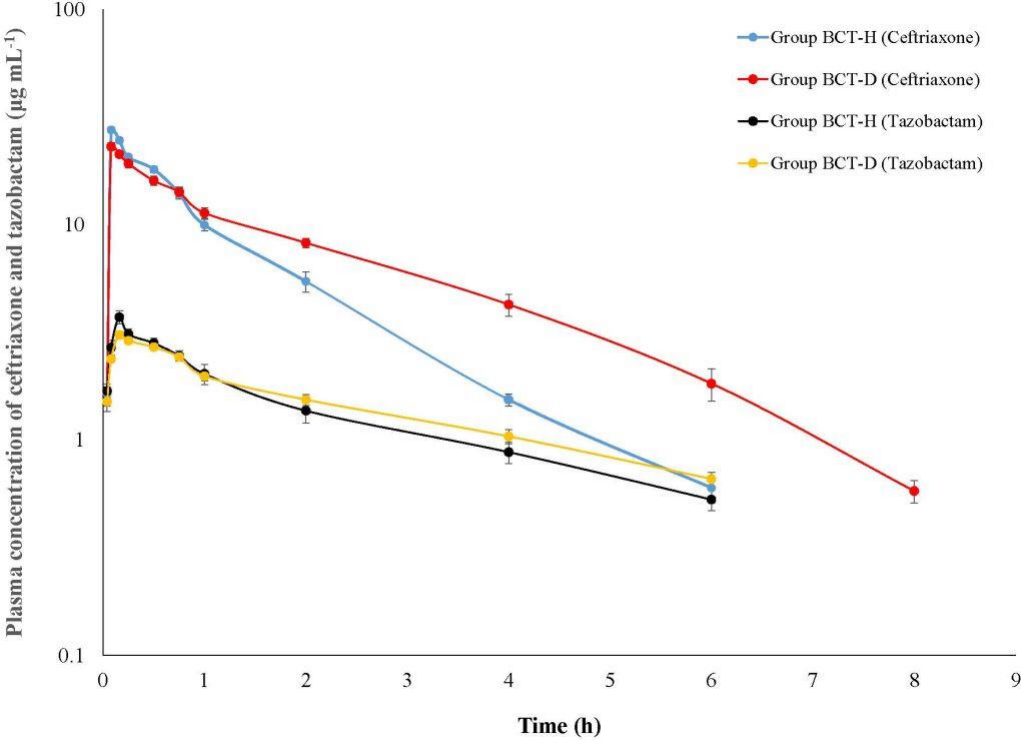
Semilogarithmic plot of plasma concentration of ceftriaxone and tazobactam in healthy (Gr BCT-H) and ESBL E. coli infected broiler birds (Gr BCT-D) following single intramuscular administration of ceftriaxone-tazobactam combination (8:1) at 28.125 mg kg^-1^.

Ceftriaxone persisted for a longer duration of 8 h in BCT-D birds compared to BCT-H birds (Figure 2). The elimination rate (β) of ceftriaxone is slower in the Gr BCT-D birds that corresponded with significantly higher plasma concentrations at 2, 4 and 6 h and a 1.5-fold higher AUC_0-inf_ (Table 1). Tazobactam persisted up to 6 h in BCT-D and BCT-H birds and maintained an approximate ratio of 1:8 with ceftriaxone up to 2 h. The elimination rate of tazobactam from the central compartment (*K*_el_) was significantly slower in BCT-D birds (Table 2), which also corresponds with the higher plasma concentration of tazobactam at 2, 4 and 6 h.

### Pharmacokinetics of ceftriaxone and tazobactam in healthy and ESBL E. coli infected Rode Island Red birds following single intramuscular administration of ceftriaxone-tazobactam combination (8:1)

**Figure 3.**
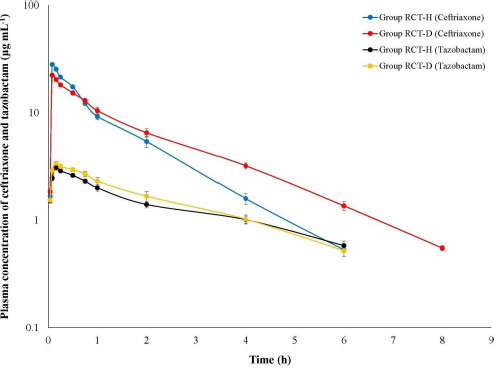
Semilogarithmic plot of plasma concentration of ceftriaxone and tazobactam in healthy (Gr RCT-H) and ESBL *E. coli* infected Rhode Island Red birds (Gr RCT-D) following single intramuscular administration of ceftriaxone-tazobactam combination (8:1) at 28.125 mg kg^-1^.

Ceftriaxone persisted up to 8 h in RCT-D birds (Fig. 3) with a *C*_max_ value of 16.99 μg mL^-1^. The *C*_max_ value was significantly lower in RCT-D birds which corresponds with significantly higher *V*d_area_. The slower elimination rate (*β*) of ceftriaxone in RCH-D birds corresponds with the significantly higher AUC_0-inf_ (Table 3). Tazobactam persisted up to 6 h in both RCT-D and RCT-H birds and it maintained 1:8 plasma concentration ratio with ceftriaxone for a much shorter time of 0.25 h. The mean value of *K*_el_ was significantly higher in Gr RCT-D birds (0.46 ± 0.02 h^-1^) compared to Gr RCT-H birds (0.39 ± 0.02 h^-1^) (Table 4).

### Pharmacokinetics of ceftriaxone and tazobactam in healthy and ESBL E. coli infected Haringhata Black birds following single intramuscular administration of ceftriaxone-tazobactam combination (8:1)

**Figure 4.**
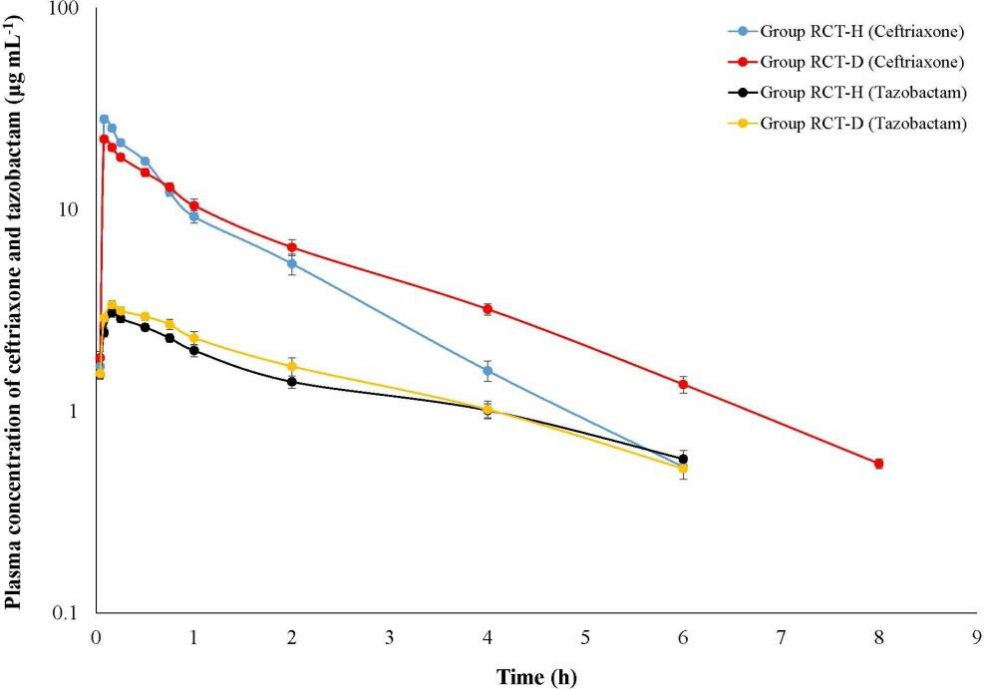
Semilogarithmic plot of plasma concentration of ceftriaxone and tazobactam in healthy (Gr HCT-H) and ESBL *E. coli* infected Haringhata Black birds (Gr HCT-D) following single intramuscular administration of ceftriaxone-tazobactam combination (8:1) at 28.125 mg kg^-1^.

Ceftriaxone persisted for a longer duration in the plasma of HCT-D group (up to 8 h) compared HCT-H group (up to 6 h) (Fig. 4). The elimination rate of ceftriaxone (*β*) is slower in Gr HCT-D group that corresponded with the higher AUC_0-inf_. The C_max_ was significantly lower in HCT-D birds, which corresponds with significantly higher *V*d_area_ values (Table 5). Tazobactam was detected up to 8 h in both HCT-H and HCT-D birds and maintained a plasma concentration of 1:8 with ceftriaxone up to 0.75 h (Figure 4). Mean values of *K*_a_, *K*_12_ and *K*_21_ were significantly higher in Gr HCT-D birds compared to Gr HCT-H birds indicating rapid absorption as well as distribution of tazobactam in diseased Haringhta Black birds (Table 6).

### Efficacy study

The efficacy of the treatment schedule was evaluated on the basis of feacal *E. coli* count though the particular counting of ESBL producing *E. coli* (TEM-1) could not be performed (Table 7). The feacal *E. coli* count reduced considerably in all the three groups following treatment with the ceftriaxone-tazobactam combination (8:1) at 28.125 mg kg^-1^ twice daily for three days. However, a significant number of *E. coli* were still present even after the recovery of the birds from diarrhoea because a large number of beneficial *E. coli* remain as a commensal organism in the intestine of birds and specific ESBL producing *E. coli* could not be counted by the present method.

### Antibody titre

**Figure 5.**
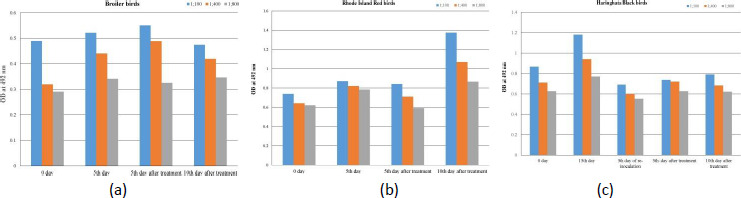
Assessment of anti-*E. coli* antibody of sensitized **(a)** Broiler birds, **(b)** Rhode Island Red birds and **(c)** Haringhata black birds.

Broiler (Fig. 5a) and Rhode Island Red (Fig. 5b) birds showed lower antibody response during the 5^th^ day of post-inoculation, but Haringhata Black birds showed higher antibody response during the 15^th^ day of first inoculation (Fig. 5c). The clinical signs of diarrhoea were observed in broiler and Rhode Island Red birds but antibody titre did not increase significantly on day 5 (during the infection period) compared to 0 day. The antibody titre was increased significantly after 5 days of treatment with ceftriaxone-tazobactam (8:1) combination in broiler and 10 days of treatment in Rhode Island Red birds. Whereas in the case of Haringhata Black birds no diarrhoea was observed after initial inoculation of ESBL *E. coli*, but antibody titre increased significantly on 15 days compared to 0 day due to natural resistance. However, following re-inoculation of these birds with ESBL *E. coli* on the 21^st^ day of first inoculation diarrhoea was observed, but treatment with ceftriaxone did not cause a significant increase in antibody titre on the 5^th^ and 10^th^ day of treatment.

The kinetic behaviour of both ceftriaxone and tazobactam followed “two compartments open model” in both healthy and ESBL *E. coli* infected broiler, Rhode Island Red and Haringhata Black birds, following single intramuscular administration of ceftriaxone-tazobactam (8:1) combination. Ceftriaxone achieved peak plasma concentration at 0.08 h and persisted up to 6 h in a healthy broiler, Rhode Island Red and Haringhata Black birds. Significantly higher values of pharmacokinetic parameters like *t*_½_ *β* and *K*_el_ corresponded with longer persistence of ceftriaxone in diarrhoeic birds up to 8 h compared to healthy birds. The plasma concentrations of ceftriaxone and tazobactam maintained an approximate ratio of 8:1 for an appreciable period (2 h) in broiler birds but for a much shorter period in Rhode Island Red (0.25 h) and Haringhata Black (0.75 h) birds post-dosing. Disposition study showed that ceftriaxone achieved a plasma concentration of 1.51 ± 0.15 μg mL^-1^, 1.84 ± 0.14 μg mL^-1^ and 1.64 ± 0.18 μg mL^-1^ in ESBL producing *E. coli* (TEM-1) induced diarrhoeic broiler, Rhode Island Red and Haringhata Black birds, respectively at 0.04 h which suggested that ceftriaxone concentration reached above the MIC value (0.12 μg mL^-1^) within 0.04 h. Moreover, the mean plasma concentrations of ceftriaxone were above the MIC value for more than 11 h (T > MIC) in all the diarrhoeic birds. Gavin et al. reported that the treatment was successful in more than 90 % of patients with non-urinary isolates when % T > MIC exceeded 40 % against ESBL producing *E. coli* [28] which was in corroboration with our findings though higher percentages of T > MIC (> 90 %) were observed. Considering these results, it was decided to administer the ceftriaxone-tazobactam combination (8:1) at 28.125 mg kg^-1^ at 12 h interval for the efficacy study. In the efficacy study, we selected b.i.d. dosing to maintain the plasma concentration of ceftriaxone within the range of 5 – 12 μg/mL (MIC 0.12 μg mL^-1^). In the ceftriaxone-tazobactam (8:1) combination, ceftriaxone is the antibacterial component and therefore, the MIC of only ceftriaxone was considered for the efficacy study. The tazobactam component of the used combination is a beta-lactamase inhibitor which is usually incorporated to prevent the destruction of ceftriaxone by beta-lactamases produced by microorganisms like ESBL *E. coli* and thereby maintaining the MIC of ceftriaxone above the target MIC. The most suitable ratio of ceftriaxone and tazobactam for effective inhibition of beta-lactamases and to increase the efficacy of ceftriaxone was reported to be 8:1 [29] and the ceftriaxone-tazobactam 8:1 combination is available commercially for effective treatment of susceptible infections. During experimental induction of infection with oral inoculation of ESBL producing *E. coli* sub-culture (56 × 10^8^ c.f.u. mL^-1^), broilers birds showed severe diarrhea with higher frequency; the major clinical sign of ESBL producing *E. coli* infection on 7^th^ day whereas moderate diarrhoea was manifested in Rhode Island Red birds from the same day. These results were correlated with the lower antibody response of broilers and Rhode Island Red during the 5^th^ day (during the infection period) of post-inoculation. Interestingly, ceftriaxone-tazobactam combination treatment at 28.125 mg kg^-1^ body weight intramuscularly twice daily for three days exhibited increased specific antibody titre on the 5^th^ and 10^th^ day of treatment in broiler and Rhode Island Red birds, respectively. Ceftriaxone, being a beta-lactam antibiotic, might cause increased specific antibody titre in the present study in broiler, Rhode Island Red birds. The role of ceftriaxone in immune stimulation by increasing antibody titre against ESBL *E. coli* was not reported earlier and is warranted for further study. Previous studies suggested that the presence of anti-pneumococcal antibodies led to therapeutic efficacy with sub-inhibitory concentrations of β-lactam antibiotics [30] and, phagocytosis mediated by human and mouse neutrophils was increased when antibiotic-resistant pneumococcal strains were incubated with serum containing specific antibodies and sub-MIC concentrations of β-lactams [31]. Interestingly, Haringhata Black birds did not show any clinical sign following the initial challenge of ESBL producing *E. coli* (TEM-1) subculture. This result was also correlated with the higher antibody response of Haringhata Black birds during the 15^th^ day of the first inoculation. This finding strongly supports the higher resistance of Haringhata Black birds, which is an indigenous poultry breed of West Bengal available only at the northern part of North 24 Parganas and the southern part of the Nadia districts of West Bengal, India. Diarrhoea began to subside on 2^nd^ day of treatment, and a complete recovery was noticed on 3^rd^ day of treatment with ceftriaxone-tazobactam (8:1) combination in all the birds. The treatment was discontinued after 3 days of treatment due to complete recovery from diarrhoea in all the birds of three groups and no other clinical signs, including diarrhoea re-appeared during the observation period. The feacal *E. coli* count reduced significantly in all the birds following treatment with the ceftriaxone-tazobactam combination (8:1). The feacal *E. coli* count results were correlated with the lower antibody response of broilers and Rhode Island Red during the 5^th^ day of post-inoculation.

## Conclusions

The ceftriaxone-tazobactam (8:1) combination showed favourable pharmacokinetics to use this combination as an effective treatment to combat particularly ESBL producing *E. coli* infection in poultry birds by administering at 28.125 mg kg^-1^ body weight intramuscularly twice daily for three days.

## Abbreviations

A: Zero time plasma concentration intercept (distribution phase); AUC_0-inf_: Total area under the plasma concentration versus time curve; B: Zero time plasma concentration intercept (elimination phase); c.f.u.: Colony forming units; Cl_B_: Total body clearance of the drug; CLSI: Clinical and laboratory standards institute; C_max__calc: maximum plasma concentration calculated from the fitted data; CPCSEA: Committee for the purpose of control and supervision of experiments on animals; EDTA: Ethylenediaminetetraacetic acid; ESBL: Extended spectrum β lactamase; F: bioavailability; f_c_: Fraction of drug in the body that is contained in the central compartment; HPLC: High performance liquid chromatography; IAEC: Institutional animal ethical committee; K_12_: First order rate constant for transfer of drug from central compartment to peripheral compartment; K_21_: First order rate constant for transfer of drug from peripheral compartment to central compartment; K_a_: rate of distribution; K_el_: First order rate constant for drug elimination from central compartment; MIC: Minimum inhibitory concentration; MPC: Mutation prevention concentration; MRT: Mean residence time; Pd: Post dosing; PDA: Photo diode array; ppm: parts per million; PMSF: Phenylmethylsulfonyl fluoride; rpm: Revolutions per minute; T > MIC: Time above MIC; T~P: Tissue to plasma ratio; T_max__calc: maximum plasma concentration time calculated from the fitted data t_½_ K_a_: Biological half-life (distribution phase); t_½_ β: Biological half-life (elimination phase); TSA: Trypticase soy agar; TSB: Trypticase soy broth; USP: United States pharmacopoeia; Vd_area_: Apparent volume of distribution (area method); Vd_c_: Apparent volume of distribution in central compartment; Vd_ss_: Steady state volume of distribution; β: Zero time plasma concentration intercept (elimination phase).

## Figures and Tables

**Table 1.** Pharmacokinetic parameters of ceftriaxone in healthy (Gr BCT-H) and ESBL *E. coli* infected Broiler birds (Gr BCT-D) following single intramuscular administration of ceftriaxone-tazobactam combination (8:1) at 28.125 mg kg^-1^.

Kinetic Parameters	Gr BCT-H	Gr BCT-D
*A* (μg mL^-1^)	23.47^*^ ± 1.27	20.23 ± 0.86
*B* (μg mL^-1^)	23.40^*^ ± 1.25	20.15 ± 0.85
*K*_a_ (h^-1^)	42.16 ± 8.51	44.77 ± 7.29
*t*_½_ *K*_a_ (h)	0.02 ± 0.01	0.02 ± 0.00
*β* (h^-1^)	0.69^*^ ± 0.05	0.43 ± 0.02
*t*_½_ *β* (h)	1.03 ± 0.07	1.62^*^ ± 0.07
AUC_0-inf_ (μg h mL^-1^)	34.86 ± 1.35	47.39^*^ ± 2.63
*V*d_area_ (L kg^-1^)	1.09 ± 0.07	1.23 ± 0.06
*Cl*_B_ (L kg^-1^ h^-1^)	12.34^*^ ± 0.58	8.89 ± 0.49
*MRT* (h)	1.45 ± 0.08	2.35^*^ ± 0.10
*V*d_c_ (L kg^-1^)	0.54 ± 0.03	0.62^*^ ± 0.03
*V*d_ss_ (L kg^-1^)	1.14 ± 0.08	1.28 ± 0.06
*K*_12_ (h^-1^)	20.11 ± 4.23	21.79 ± 3.65
*K*_21_ (h^-1^)	21.38 ± 4.25	22.55 ± 3.63
*K*_el_ (h^-1^)	1.36^*^ ± 0.10	0.86 ± 0.04
*f*_c_	0.51 ± 0.00	0.50 ± 0.00
T~P	0.96 ± 0.01	0.98 ± 0.00
*C*_max__calc (μg mL^-1^)	21.18 ± 1.47	19.00 ± 0.89
*T*_max__calc (h)	0.16 ± 0.03	0.15 ± 0.01
*T* > MIC	-	11.08 h (92 %)
*F*	0.14	0.12

Mean values in a raw bearing superscript

^*^ vary significantly (P < 0.05); [n=6]

**Table 2.** Pharmacokinetic parameters of tazobactam in healthy (Gr BCT-H) and ESBL *E. coli* infected broiler birds (Gr BCT-D) following single intramuscular administration of ceftriaxone-tazobactam combination (8:1) at 28.125 mg kg^-1^.

Kinetic Parameters	Gr BCT-H	Gr BCT-D
*A* (μg mL^-1^)	3.37 ± 0.22	3.06 ± 0.11
*B* (μg mL^-1^)	3.42 ± 0.21	3.10 ± 0.13
*K*_a_ (h^-1^)	14.53 ± 3.12	11.91 ± 1.39
*t*_½_ *K*_a_ (h)	0.06 ± 0.01	0.06 ± 0.01
*β* (h^-1^)	0.24^*^ ± 0.01	0.19 ± 0.01
*t*_½_ *β* (h)	2.99 ± 0.18	3.72^*^ ± 0.24
AUC_0-inf_ (μg h mL^-1^)	15.02 ± 1.30	16.72 ± 0.74
*V*d_area_ (L kg^-1^)	0.96 ± 0.06	1.04 ± 0.05
*Cl*_B_ (L kg^-1^ h^-1^)	3.76 ± 0.34	3.32 ± 0.16
*MRT* (h)	4.38 ± 0.26	5.41^*^ ± 0.38
*V*d_c_ (L kg^-1^)	3.76 ± 0.22	4.09 ± 0.17
*V*d_ss_ (L kg^-1^)	7.89 ± 0.45	8.51 ± 0.28
*K*_12_ (h^-1^)	6.90 ± 1.56	5.66 ± 0.71
*K*_21_ (h^-1^)	7.40 ± 1.55	6.06 ± 0.68
*K*_el_ (h^-1^)	0.46* ± 0.03	0.37 ± 0.02
*f*_c_	0.51 ± 0.01	0.51 ± 0.00
T~P	0.95 ± 0.02	0.96 ± 0.01
*C*_max__calc (μg mL^-1^)	3.09 ± 0.17	2.83 ± 0.09
*T*_max__calc (h)	0.36 ± 0.05	0.39 ± 0.03
*F*	0.16	0.15

Mean values in a raw bearing superscript

^*^ vary significantly (P < 0.05); [n=6]

**Table 3.** Pharmacokinetic parameters of ceftriaxone in healthy (Gr RCT-H) and ESBL *E. coli* infected Rhode Island Red birds (Gr RCT-D) following single intramuscular administration of ceftriaxone-tazobactam combination (8:1) at 28.125 mg kg^-1^.

Kinetic Parameters	Gr RCT-H	Gr RCT-D
*A* (μg mL^-1^)	22.73^*^ ± 0.31	18.34 ± 1.00
*B* (μg mL^-1^)	22.65^*^ ± 0.29	18.28 ± 1.00
*K*_a_ (h^-1^)	32.46 ± 4.48	33.78 ± 4.24
*t*_½_ *K*_a_ (h)	0.02 ± 0.00	0.02 ± 0.00
*β* (h^-1^)	0.70^*^ ± 0.05	0.44 ± 0.01
*t*_½_ *β* (h)	1.01 ± 0.06	1.57^*^ ± 0.05
AUC_0-inf_ (μg h mL^-1^)	33.66 ± 1.93	41.70^*^ ± 1.67
*V*d_area_ (L kg^-1^)	1.10 ± 0.02	1.43^*^ ± 0.11
*Cl*_B_ (L kg^-1^ h^-1^)	12.86^*^ ± 0.86	10.45 ± 0.59
*MRT* (h)	1.43 ± 0.08	2.25^*^ ± 0.07
*V*d_c_ (L kg^-1^)	0.55 ± 0.01	0.69^*^ ± 0.04
*V*d_ss_ (L kg^-1^)	1.16 ± 0.02	1.43^*^ ± 0.10
*K*_12_ (h^-1^)	15.24 ± 2.21	16.27 ± 2.12
*K*_21_ (h^-1^)	16.55 ± 2.25	17.08 ± 2.12
*K*_el_ (h^-1^)	1.37^*^ ± 0.10	0.88 ± 0.03
*f*_c_	0.5110^*^ ± 0.00	0.5063 ± 0.00
T~P	0.96 ± 0.01	0.98^*^ ± 0.00
*C*_max__calc (μg mL^-1^)	20.21^*^ ± 0.38	16.99 ± 1.02
*T*_max__calc (h)	0.17 ± 0.02	0.17 ± 0.02
*T* > MIC	-	11.16 h (92.67 %)
*F*	0.14	0.11

Mean values in a raw bearing superscript

^*^ vary significantly (P < 0.05)

**Table 4.** Pharmacokinetic parameters of tazobactam in healthy (Gr RCT-H) and ESBL *E. coli* infected Rhode Island Red birds (Gr RCT-D) following single intramuscular administration of ceftriaxone-tazobactam combination (8:1) at 28.125 mg kg^-1^.

Kinetic Parameters	Gr RCT-H	Gr RCT-D
*A* (μg mL^-1^)	2.96 ± 0.17	3.48^*^ ± 0.19
*B* (μg mL^-1^)	3.07 ± 0.15	3.47 ± 0.19
*K*_a_ (h^-1^)	13.28 ± 2.59	19.32 ± 3.19
*t*_½_ *K*_a_ (h)	0.07 ± 0.02	0.04 ± 0.01
*β* (h^-1^)	0.20 ± 0.01	0.23 ± 0.01
*t*_½_ *β* (h)	3.45 ± 0.19	3.03 ± 0.18
AUC_0-inf_ (μg h mL^-1^)	15.55 ± 1.00	15.37 ± 1.30
*V*d_area_ (L kg^-1^)	1.05 ± 0.05	0.80 ± 0.15
*Cl*_B_ (L kg^-1^ h^-1^)	3.56 ± 0.27	3.54 ± 0.26
*MRT* (h)	4.96^*^ ± 0.28	4.28 ± 0.23
*V*d_c_ (L kg^-1^)	4.19^*^ ± 0.20	3.65 ± 0.18
*V*d_ss_ (L kg^-1^)	8.96^*^ ± 0.57	7.49 ± 0.35
*K*_12_ (h^-1^)	6.31 ± 1.33	9.33 ± 1.61
*K*_21_ (h^-1^)	6.78 ± 1.26	9.76 ± 1.59
*K*_el_ (h^-1^)	0.39 ± 0.02	0.46^*^ ± 0.02
*f*_c_	0.52 ± 0.01	0.51 ± 0.00
T~P	0.93 ± 0.04	0.98 ± 0.01
*C*_max__calc (μg mL^-1^)	2.79 ± 0.12	3.21^*^ ± 0.15
*T*_max__calc (h)	0.38 ± 0.05	0.30 ± 0.03
*F*	0.15	0.17

Mean values in a raw bearing superscript

^*^ vary significantly (P < 0.05); [n=6]

**Table 5.** Pharmacokinetic parameters of ceftriaxone in healthy (Gr HCT-H) and ESBL *E. coli* infected Haringhata Black birds (Gr HCT-D) following single intramuscular administration of ceftriaxone-tazobactam combination (8:1) at 28.125 mg kg^-1^.

Kinetic Parameters	Gr HCT-H	Gr HCT-D
*A* (μg mL^-1^)	23.47^*^ ± 1.27	18.41 ± 0.94
*B* (μg mL^-1^)	23.40^*^ ± 1.25	18.34 ± 0.92
*K*_a_ (h^-1^)	42.16 ± 8.51	37.00 ± 4.44
*t*_½_ *K*_a_ (h)	0.02 ± 0.01	0.02 ± 0.00
*β* (h^-1^)	0.69^*^ ± 0.05	0.44 ± 0.02
*t*_½_ *β* (h)	1.03 ± 0.07	1.59^*^ ± 0.07
AUC_0-inf_ (μg h mL^-1^)	34.86 ± 1.35	42.32^*^ ± 2.13
*V*d_area_ (L kg^-1^)	1.09 ± 0.07	1.39^*^ ± 0.08
*Cl*_B_ (L kg^-1^ h^-1^)	12.34^*^ ± 0.58	10.19 ± 0.57
*MRT* (h)	1.45 ± 0.08	2.29^*^ ± 0.10
*V*d_c_ (L kg^-1^)	0.54 ± 0.03	0.69^*^ ± 0.03
*V*d_ss_ (L kg^-1^)	1.14 ± 0.08	1.41^*^ ± 0.07
*K*_12_ (h^-1^)	20.11 ± 4.23	17.88 ± 2.23
*K*_21_ (h^-1^)	21.38 ± 4.25	18.68 ± 2.21
*K*_el_ (h^-1^)	1.36^*^ ± 0.10	0.87 ± 0.04
*f*_c_	0.51 ± 0.00	0.51 ± 0.00
T~P	0.96 ± 0.01	0.98 ± 0.00
*C*_max__calc (μg mL^-1^)	21.18^*^ ± 1.47	17.12 ± 0.94
*T*_max__calc (h)	0.16 ± 0.03	0.17 ± 0.02
*T* > MIC	-	11.24 h (93.3 %)
*F*	0.13	0.10

Mean values in a raw bearing superscript

^*^ vary significantly (P < 0.05); [n=6]

**Table 6.** Pharmacokinetic parameters of tazobactam in healthy (Gr HCT-H) and ESBL *E. coli* infected Haringhata Black birds (Gr HCT-D) following single intramuscular administration of ceftriaxone-tazobactam combination (8:1) at 28.125 mg kg^-1^.

Kinetic Parameters	Gr HCT-H	Gr HCT-D
*A* (μg mL^-1^)	3.06 ± 0.11	3.22 ± 0.10
*B* (μg mL^-1^)	3.10 ± 0.13	3.20 ± 0.11
*K*_a_ (h^-1^)	11.91 ± 1.39	18.77^*^ ± 1.73
*t*_½_ *K*_a_ (h)	0.06^*^ ± 0.01	0.04 ± 0.00
*β* (h^-1^)	0.19 ± 0.01	0.21 ± 0.01
*t*_½_ *β* (h)	3.72 ± 0.24	3.30 ± 0.17
AUC_0-inf_ (μg h mL^-1^)	16.72 ± 0.74	15.40 ± 0.80
*V*d_area_ (L kg^-1^)	1.04 ± 0.05	0.99 ± 0.03
*Cl*_B_ (L kg^-1^ h^-1^)	3.32 ± 0.16	3.50 ± 0.17
*MRT* (h)	5.41 ± 0.38	4.82 ± 0.21
*V*d_c_ (L kg^-1^)	4.09 ± 0.17	3.91 ± 0.13
*V*d_ss_ (L kg^-1^)	8.51 ± 0.28	8.00 ± 0.25
*K*_12_ (h^-1^)	5.66 ± 0.71	9.09^*^ ± 0.87
*K*_21_ (h^-1^)	6.06 ± 0.68	9.46^*^ ± 0.86
*K*_el_ (h^-1^)	0.37 ± 0.02	0.42 ± 0.02
*f*_c_	0.51 ± 0.00	0.50 ± 0.00
T~P	0.96 ± 0.01	0.98 ± 0.00
*C*_max__calc (μg mL^-1^)	2.83 ± 0.09	3.00 ± 0.09
*T*_max__calc (h)	0.39^*^ ± 0.03	0.30 ± 0.02
*F*	0.14	0.15

Mean values in a raw bearing superscript

^*^ vary significantly (P < 0.05); [n=6]

**Table 7.** Mean values of faecal *E. coli* colony count in broilers, Rhode Island Red and Haringhata black birds during diarrhoea (before the start of treatment) and after three days of treatment with ceftriaxone-tazobactam combination at 28.125 mg kg^-1^ bid intramuscularly for 3 days. [n=6]

Groups	During diarrhoea (c.f.u. gm^-1^)	After treatment (c.f.u. gm^-1^)
Gr BE	25 × 10^9^	19 × 10^7^
Gr RE	31 × 10^9^	25 × 10^6^
Gr HE	45 × 10^9^	23 × 10^5^

## References

[1] Department of Animal Husbandry, Dairying & Fisheries, Ministry of Agriculture & Farmers Welfare, Government of India. National action plan for egg & poultry- 2022 for doubling farmers’ income by 2022. https://www.dahd.nic.in/sites/default/filess/Seeking%20Comments%20on%20National%20Action%20Plan-%20Poultry-%202022%20by%2012-12-2017.pdf.

[2] Barnes H.J. Gross W.B. Colibacillosis In Diseases of Poultry, Calnek B.K. Barnes H.J. Beard C.W., Eds., Iowa State University Press, Mosby-Wolfe, (1997), p. 138–144.

[3] Rosenberger J.K. Fries P.A. Cloud S.S. *In vitro* and *in vivo* characterization of avian *Escherichia coli*. III. Immunization. Avian Dis. 29 (1985) 1108–1117. 3914273

[4] Melamed D. Letner G. Heller E.D. A vaccine against avian colibacillosis based on ultrasonic inactivation of *Escherichia coli*. Avian Dis. 35 (1991) 17–22. 2029251

[5] Dhillon A.S. Jack O.K. Two outbreaks of colibacillosis in commercial caged layers. Avian Dis. 40 (1996) 742–746. 8883810

[6] Blanco M. Blanco J.E. Mora A. Blanco J. *Escherichia coli* septicemicos aviares: serotipos, factores de virulencia, resistencia a antibiotics y desarrollo de vacunas. Medicina Veterinaria 13 (1996) 525–537.

[7] Casellas J.M. South America: A different continent, different ESBLs. J. Antimicrob. Chemother. 44 (A) (1999) 16.

[8] Poirel L. LeThomas I. Naas T. Karim A. Nordmann P. Biochemical sequence analyses of GES-1, a novel class A Extended-Spectrum Beta- Lactamase, and the Class 1 Integron In52 from *Klebsiella pneumoniae*. Antimicrob. Agents Chemother. 44 (2000) 622–632. https://doi.org/10.1128/AAC.44.3.622-632.2000. 10.1128/AAC.44.3.622-632.2000 10681329 PMC89737

[9] Samanta I. Joardar S.N. Das P.H. Das P. Sar T.K. Dutta T.K. Bandyopadhyay S. Batabyal S. Isore D.P. Virulence repertoire, characterization, and antibiotic resistance pattern analysis of *Escherichia coli* isolated from backyard layers and their environment in India. Avian Dis. 58(1) (2014) 39-45. https://doi.org/10.1637/10586-052913-Reg.1. 10.1637/10586-052913-Reg.1 24758111

[10] Chowdhury M. Bardhan R. Pal S. Banerjee A. Batabyal K. Joardar S.N. Mandal G.P. Bandyopadhyay S. Dutta T.K. Sar T.K. Samanta I. Comparative occurrence of ESBL/AmpC beta-lactamase-producing *Escherichia coli* and Salmonella in contract farm and backyard broilers. Lett. Appl. Microbiol. 74 (2021) 53-62. https://doi.org/10.1111/lam.13581. 10.1111/lam.13581 34618368

[11] Hussain A. Shaik S. Ranjan A. Nandanwar N. Tiwari S.K. Majid M. Baddam R. Qureshi I.A. Semmler T. Wieler L.H. Islam M.A. Chakravortty D. Ahmed N. Risk of transmission of antimicrobial resistant *Escherichia coli* from commercial broiler and free-range retail chicken in India. Front. Microbiol. 8 (2017) 2120. https://doi.org/10.3389/fmicb.2017.02120. 10.3389/fmicb.2017.02120 29180984 PMC5694193

[12] Neu H.C. Meropolol N.J. Fu K.P. Antibacterial activity of ceftriaxone (Ro 13-9904) and a β-lactamase stable cephalosporin. Antimicrob. Agents Chemother. 19 (1981) 414–423. https://doi.org/10.1128/AAC.19.3.414. 10.1128/AAC.19.3.414 6972729 PMC181447

[13] Li T. Qiao G.L. Hu G.Z. Meng F.D. Qiu Y.S. Zhang X.Y. Guo W.X. Yie H.L. Li S.F. Li S.Y. Comparative plasma and tissue pharmacokinetics and drug residue profiles of different chemotherapeutants in fowls and rabbits. J. Vet. Pharmacol. Ther. 18(4) (1995) 260-273. https://doi.org/10.1111/j.1365-2885.1995.tb00590.x. 10.1111/j.1365-2885.1995.tb00590.x 8583539

[14] Kumar P. Singh K.P. Ahuja V. Ahmed A.H. Pharmacokinetics of ceftriaxone following single dose i.v. and i.m. administration in layer birds. Journal of Veterinary Pharmacology and Toxicology 8(1-2) (2010) 10-12.

[15] Queenan A.M. Foleno B. Gownley C. Effects of inoculum and β-lactamase activity in AmpC- and extended-Spectrum β-lactamase (ESBL)-producing *Escherichia coli* and *klebsiella pneumoniae* clinical isolates tested by using NCCLS ESBL methodology. J. Clin. Microbiol. 42 (2004) 269–275. https://doi.org/10.1128/JCM.42.1.269-275.2004. 10.1128/JCM.42.1.269-275.2004 14715764 PMC321709

[16] Payasi A. Kumar S. Chaudhary M.A. Comparative study of sulbactomax versus ceftriaxone and betalactamase inhibitor and their effect on mutant prevention in ESBL producing organisms. International Journal of Drug Development and Research 3(3) (2011) 366-371.

[17] Payne D.J. Cramp R. Winstanley D.J. Knowles D.J Comparative activities of clavulanic acid, sulbactam, and tazobactam against clinically important β-lactamases. Antimicrob. Agents Chemother. 38(4) (1994) 767–772. https://doi.org/10.1128/AAC.38.4.767. 10.1128/AAC.38.4.767 8031044 PMC284540

[18] Mithin U.C. Buragohain R. Sar T.K. Mandal T.K. Disposition kinetics of ceftriaxone and tazobactam in broiler, Rhode Island Red and Haringhata Black poultry following single dose intramuscular administration. International Journal of Modern Pharmaceutical Research 4(6) (2020).

[19] Mithin U.C. Buragohain R. Das P.K. Mandal T.K. Hansda R.N. Joardar S.N. Samanta I. Sar T.K. Monitoring of liver markers in poultry during post inoculation of extended spectrum β lactamases producing *Escherichia coli* and after treatment by ceftriaxone-tazobactam combination. Indian J. Anim. Hlth. 59(2) (2020) 237-242. https://doi.org/10.36062/ijah.59.2SPL.2020.237-242. 10.36062/ijah.59.2SPL.2020.237-242

[20] Clinical and Laboratory Standards Institute. Performance standards for antimicrobial susceptibility testing: Twenty-third informational supplement M100-S23. CLSI, Wayne, PA, USA, 2013.

[21] Sar T.K. Patra P.H. Dash J.R. Mandal T.K. Pharmacokinetic interaction of intramammary ceftriaxone and oral polyherbal drug (Fibrosin) in goats. Drug Metabol. Drug Interact. 24(4) (2011) 191-196. https://doi.org/10.1515/DMDI.2011.019. 10.1515/DMDI.2011.019 22098637

[22] Ocampo A.P. Hoyt K.D. Wadgaonkar N. Carver A.H. Puglisi C.V. Determination of tazobactam and piperacillin in human plasma, serum, bile and urine by gradient elution Reversed-Phase HPLC. J. Chromatogr. 496 (1089) 167-179. https://doi.org/10.1016/S0378-4347(00)82563-3. 10.1016/S0378-4347(00)82563-3 2556418

[23] Tamboli S.R. Patil D.D. RP-HPLC method for simultaneous estimation of cefepime hydrochloride and tazobactam sodium in bulk and pharmaceuticals. J. Chem. 208057 (2013) 6 pages. https://doi.org/10.1155/2013/208057. 10.1155/2013/208057

[24] Baggot J.D.. The Basis of Veterinary Clinical Pharmacology, W.B. Saunders Co., Philadelphia, London, 1977.

[25] Quinn P.J. Carter M.E. Markey B.K. Carter G.R. Clinical Veterinary Microbiology, Wolf publishing, London, UK, 1994.

[26] Mockett A.P.A. Cook J.K.A. Huggins M.B. Maternally derived antibody to infectious bronchitis virus: its detection in chick trachea and serum and its role in protection. Avian Pathol. 16 (1987) 407–416. https://doi.org/10.1080/03079458708436391. 10.1080/03079458708436391 18766630

[27] Choi K.H. Maheswaran S.K. Felice L.J. Characterization of outer membrane protein enriched extracts from *Pasteurella multocida* isolated from turkeys. Am. J. Vet. Res. 50 (1989) 676-683. 2729713

[28] Gavin P.J. Suseno M.T. Thomson R.B. Gaydos J.M. Pierson C.L. Halstead D.C. Aslanzadeh J. Brecher S. Rotstein C. Brossette S.E. Peterson L.R. Clinical correlation of the CLSI susceptibility breakpoint for piperacillin-tazobactam against extended-spectrum-β-lactamase-producing *Escherichia coli* and *Klebsiella* species. Antimicrob. Agents Chemother. 50(6) (2006) 2244–2247. https://doi.org/10.1128/AAC.00381-05. 10.1128/AAC.00381-05 16723596 PMC1479103

[29] Georgopoulos A. Buxbaum A. Graninger W. Efficacy of β-lactam and inhibitor combinations in a diffusion chamber model in rabbits. J. Antimicrob. Chemother. 43(4) (1999) 497–501. https://doi.org/10.1093/jac/43.4.497. 10.1093/jac/43.4.497 10350378

[30] Casal J. Aguilar L. Jado I. Yuste J. Giménez M.J. Prieto J. Fenoll A. Effects of specific antibodies against *Streptococcus pneumoniae* on pharmacodynamic parameters of beta-lactams in a mouse sepsis model. Antimicrob. Agents Chemother. 46 (2002) 1340–1344. https://doi.org/10.1128/AAC.46.5.1340-1344.2002. 10.1128/AAC.46.5.1340-1344.2002 11959566 PMC127147

[31] Cafini F. Yuste J. Giménez M.J. Sevillano D. Aguilar L. Alou L. Ramos-Sevillano E. Torrico M. González N. García E. Coronel P. Prieto J. Enhanced in vivo activity of cefditoren in pre-immunized mice against penicillin-resistant *S. pneumoniae* (serotypes 6B, 19F and 23F) in a sepsis model. PLoS One. (2010) 5:e12041. https://doi.org/10.1371/journal.pone.0012041. 10.1371/journal.pone.0012041 PMC2919394 20706584

